# A scoping review of the associations between mental health and factors related to HIV acquisition and disease progression in conflict-affected populations

**DOI:** 10.1186/s13031-018-0156-y

**Published:** 2018-06-01

**Authors:** Erica Koegler, Caitlin E. Kennedy

**Affiliations:** 1 Department of Health Sciences, University of Missouri, 512 Clark Hall, Columbia, MO 65211 USA; 2 Department of International Health, Johns Hopkins Bloomberg School of Public Health, 615 N. Wolfe Street Room E5547 Baltimore, Baltimore, MD 21205 USA

**Keywords:** Mental health, HIV, Conflict settings, Depression, Anxiety, PTSD

## Abstract

The association between poor mental health and factors related to HIV acquisition and disease progression (also referred to as HIV-related factors) may be stronger among conflict-affected populations given elevated rates of mental health disorders. We conducted a scoping review of the literature to identify evidence-based associations between mental health (depression, anxiety, and post-traumatic stress disorder [PTSD]) and factors related to HIV acquisition and progression in conflict-affected populations. Five electronic databases were searched on October 10, 2014 and updated on March 7, 2017 to identify peer-reviewed publications presenting primary data from January 1, 1994 to March 7, 2017. Articles were included if: 1) depression, anxiety, and/or PTSD was assessed using a validated scale, 2) HIV or HIV-related factors were a primary focus, 3) quantitative associations between depression/anxiety/PTSD and HIV or HIV-related factors were assessed, and 4) the study population was conflict-affected and from a conflict-affected setting. Of 714 citations identified, 33 articles covering 110,818 participants were included. Most were from sub-Saharan Africa (*n* = 25), five were from the USA, and one each was from the Middle East, Europe, and Latin America. There were 23 cross-sectional, 3 time-series, and 7 cohort studies. The search identified that mental health has been quantitatively associated with the following categories of HIV-related factors in conflict-affected populations: markers of HIV risk, HIV-related health status, sexual risk behaviors, and HIV risk exposures (i.e. sexual violence). Further, findings suggest that symptoms of poor mental health are associated with sexual risk behaviors and HIV markers, while HIV risk exposures and health status are associated with symptoms of poor mental health. Results suggest a role for greater integration and referrals across HIV and mental health programs for conflict-affected populations.

**Electronic supplementary material:**

The online version of this article (10.1186/s13031-018-0156-y) contains supplementary material, which is available to authorized users.

## Background

The relationship between mental health and HIV acquisition and disease progression (also referred to as HIV-related factors) is bi-directional. Having symptoms of post-traumatic stress disorder (PTSD), depression, and/or anxiety has been linked to HIV risk factors in various populations both prospectively [1] and cross-sectionally [2–6]. Being HIV-positive physiologically, psychologically, and socially increases risk for neuropsychiatric conditions [7]. In this paper we consider a broad range of factors associated with HIV acquisition and disease progression, such as markers of HIV risk, HIV-related health status, sexual risk behaviors, and other potential HIV risk exposures not under individual control (i.e. sexual violence).

As conflict-affected populations often have elevated rates of PTSD, depression, and anxiety [8–12], the association between poor mental health and risk for HIV acquisition and disease progression may be stronger among these populations. Conflict can shape population movements, opportunities for sexual partnering, and mortality patterns in ways that might increase or decrease HIV prevalence [13, 14]. Epidemiological evidence suggests elevated HIV prevalence in the five years prior to conflict, but an overall decrease in HIV prevalence during and just after conflict [15, 16]. However, vulnerable populations may remain at elevated risk for HIV acquisition during political and socioeconomic instability [14, 17]. A seminal paper discussing population vulnerability to HIV transmission in conflict-affected settings discusses health factors but does not detail the ways poor mental health can impact population vulnerability to HIV [13]. Poor mental health, conflict, and being HIV positive are independently related to morbidity and mortality. Co-occurrence of these factors can contribute to increased vulnerability to morbidity and mortality.

Other reviews that have examined associations between mental health and HIV risk behaviors or care and treatment programs have focused on migrant populations [18] and populations from developing countries [18, 19]. It is yet unknown how the vulnerabilities of poor mental health and factors related to HIV acquisition and disease progression operate in conflict-affected populations. Understanding how mental health is associated with HIV acquisition and disease progression in conflict-affected populations can inform program and policy work in these settings. The aim of our study was to conduct a scoping review of the literature to identify evidence-based associations between common mental health conditions (depression, anxiety, and PTSD) and factors related to HIV acquisition and disease progression in conflict-affected populations. We sought to understand the bi-directional associations between these mental health conditions and various measures of HIV-related factors, and to examine the strength and directionality of associations to offer suggested directions for future research, policy, and interventions.

## Methods

Peer-reviewed publications that presented primary data from January 1, 1994 to March 7, 2017 were included in this review if they met the following inclusion criteria: 1) one or more of three common mental health conditions (depression, anxiety, PTSD) was a primary or substantive focus of the article and was assessed using a validated scale, 2) HIV serostatus or factors related to HIV acquisition and disease progression (defined below) was a primary or substantive focus of the article, 3) the quantitative relationship between HIV or HIV-related factors and the mental health condition(s) was discussed, and 4) the study reported that participants were conflict-affected and from a conflict-affected setting. All age groups were included in this review. All study designs were considered as long as the four inclusion criteria were met. Articles were excluded if they measured HIV-related factors on a war events scale but did not present data for the relationship between the HIV-related factor alone and mental health measures. This review was conducted following PRISMA guidelines [20].

### Definition of terms

We sought to illuminate the ways a broad range of factors related to HIV acquisition and disease progression have been examined in relationship to mental health. Therefore, we defined factors related to HIV acquisition and disease progression to include factors such as: markers of HIV risk (i.e. sexually transmitted infections (STIs)); HIV-related health status (i.e. HIV seropositive status, CD4 count); sexual risk behaviors (e.g. unprotected sex, multiple sexual partners, exchange sex, etc.); and other potential HIV exposures not under individual control (i.e. sexual assault). A range of factors related to HIV were included in order to provide a comprehensive understanding of how researchers have quantitatively examined the relationships between specific mental health disorders and HIV-related factors.

Conflict-affected settings were defined according to UNESCO as areas with ‘explosive’ (over 200 battle-related deaths in a year) or ‘protracted’ (over 1000 battle-related deaths over ten years) events [21]. Both active conflict and post-conflict settings were included. Since not all populations in conflict-affected countries are directly affected by conflict, the study population had to be affected by conflict and described as such by the article’s authors. All combat-affected populations were considered for inclusion, both combatants and civilians. Conflict-affected populations across the economic spectrum were considered for inclusion to examine the relationship between mental health and HIV-related factors in a variety of economic situations.

### Search strategy

Five electronic databases (PubMed, PsycINFO, SCOPUS, CINAHL, and EMBASE) were searched first on October 10, 2014 and updated on March 7, 2017. Search terms included combinations of terms for mental health, HIV risk, and conflict-affected settings (Additional file 1). We also searched reference lists of included articles and hand searched the table of contents of *Conflict and Health* and *Medicine Conflict and Survival*. Only articles with an abstract in English were screened.

### Data extraction and management

Articles were screened and data extracted by one reviewer (EK), with uncertainty resolved through discussion with a second reviewer (CK). A third reviewer verified all data presented in Tables 1 and 2. First, titles and abstracts identified through the search strategy were screened. Full text articles were obtained for all selected abstracts. An eligibility form was completed to determine final study selection. Data were extracted using a standardized data extraction spreadsheet. For each included study the following information was extracted where applicable: citation; location, setting and target group; study design; sample size; age range; gender; random or non-random selection of participants; length of follow up; outcome measures; comparison groups; effect sizes; confidence intervals; significance levels; measures of HIV risk, mental health conditions and measures; funding source; and study limitations. To assess study quality, we extracted data on study design, sampling strategy, sample size, and participant characteristics, presented in Table 1. These factors were then considered in relation to study quality as presented in the results and discussion. We did not conduct meta-analysis due to the diversity of populations, study designs, and measured outcomes.

## Results

**Fig. 1:**
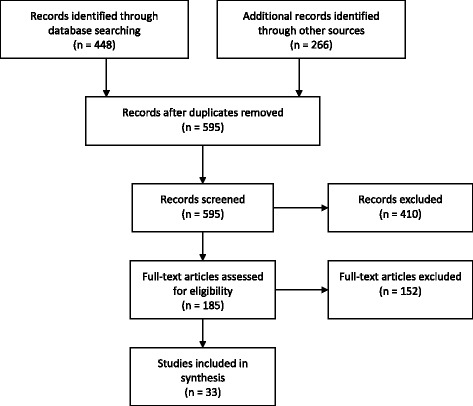
Flow Diagram of review. This flow diagram depicts the search and screening process for the review. Of the 714 records identified through database searching and other sources, 33 articles were ultimately included in this review

Of 714 citations identified through the search strategy, 33 publications were included in this review (Tables 1 and 2). Figure 1 presents a flowchart of the search and screening process. Eighteen articles were identified via database searching, thirteen through reference searching, and two through searching journal table of contents.

**Table 1:** Description of included studies

Primary author and Year	Country	Study design	Sampling strategy	Sample size and participation rate	Participant characteristics
Mental health and HIV serostatus/HIV-related outcomes					
Adedimeji et al., 2015	Rwanda	Cross-sectional Baseline data from 2005 RWISA prospective cohort	Non random selection HIV+ and HIV- women approximately 50% of whom experienced rape during the genocide	*N* = 928 99% of *N* = 936 included	Women over age 15 who experienced the 1994 genocide, 76% HIV+ 20.5% < 30 years 48.4% 30–40 years 31.1% 40+ years 100% female
Adler et al., 2011	USA	Time-series Time 1: 4 months after return from deployment Time 2: 4 months later	Non random selection Part of a larger study on post deployment transition	*N* = 647 39% of *N* = 1651 included who completed both assessments	Active duty USA soldiers in a brigade combat team who had returned from a 15-month deployment in Iraq Age not reported 96% male 4% female
Kinyanda et al., 2012	Uganda	Cross-sectional Nested in study on HIV-related psychiatric & psychosocial vulnerabilities in war-affected community	Random selection Multistage sampling to include vulnerable and non-vulnerable individuals	*N* = 1560 98.5% of *N* = 1584 included who completed the interview	Vulnerable (widows, orphans, single mothers) and non-vulnerable individuals in a war-affected community Aged 15 years and older 56% were aged between 18 and 44 years 43% male 57% female
Kinyanda et al., 2016	Uganda	Cross-sectional Nested in study addressing HIV-related psychiatric and psychosocial vulnerabilities in the war-affected community	Random sampling Multistage sampling for representative sample of vulnerable and non-vulnerable individuals	*N* = 1110 71.2% with complete data included of *N* = 1560	Vulnerable (widowed, divorced, orphan, suffered torture, mental illness, etc.) and non-vulnerable individuals in a war-affected community Aged 15 years and older 56% were aged between 18 and 44 years 43% male 57% female
Malamba et al., 2016	Uganda	Cross-sectional Baseline data from a longitudinal cohort study to determine HIV prevalence and risk factors to inform program development	Random selection Two-stage stratified sampling for representative sample	*N* = 2388 97.5% who had HIV results included of *N* = 2449 consenting individuals	Conflict affected individuals aged 13–49 29.1% 13–19 years 20.2% 20–24 years 19.6% 25–29 years 12.4% 30–34 years 18.6% 35+ years 40% male 60% female
Svetlicky et al., 2010	Lebanon	Cross-sectional Collected 6 months post-conflict, collected for 4 months.	Non random selection Combat reserve soldiers who sought treatment in the Combat Reaction Unit in the wake of the Second Lebanon War	*N* = 180 65.7% of *N* = 274 included who completed questionnaires	Mean age = 29.95 years (SD = 5.82; range = 20 to 54 years). 100% male Most were Israeli-born (82.8%)
Talbot et al., 2013	Rwanda	Time-series Collected at baseline, 5, 9, and 12 months	Random selection Orphans selected via random number generation from a list of all eligible orphans enrolled in program	*N* = 120 95% of *N* = 120 completed all 4 assessments; all participants were included in analysis	94% were orphaned from the genocide Mean age = 18 years (range 15–25) Male 47% Female 53%
B.E. Cohen et al., 2012	USA	Retrospective cohort From a roster of all USA veterans from 2 operations	Non random selection Separated USA veterans who were new users of Department of Veterans Affairs healthcare	*N* = 71,504	Veterans of Operations Enduring and Iraqi Freedom Mean age = 28.5 to 29.5 100% female
Sexual violence and mental health outcomes					
Amone P’Olak et al., 2013	Uganda	Cross-sectional Baseline data nested in a before and after study	Random selection War-affected youth who had been abducted and lived in rebel captivity for at least 6 months	*N* = 539 83% of *N* = 650 who were invited to the study	Aged between 18 and 25 years 61% male 39% female 86% Acholi ethnic group
Roberts et al., 2008	Uganda	Cross-sectional	Random selection Multi-stage cluster sampling of camps, administrative zones, and individuals	*N* = 1210	Adults living in camps for internally displaced persons Mean age = 35.3 years 40% male 60% female 91% Acholi ethnic group
Nakimuli-Mpungu et al., 2013	Uganda	Time series Collected at baseline, 3, and 6 months	Non random selection Analysis included only adults with a history of war-related traumatic experiences	*N* = 375 59% of *N* = 631 included who were present for at least 2 visits	Demographic data reported all patients *N* = 2868, many of whom were not included in the main analysis Mean age adult men = 34.5 Mean age adult women = 37.3 47% male 53% female
Okello et al., 2007	Uganda	Case control Cross- sectional, unmatched Cases were formerly abducted youth Controls were non abducted youth	Random selection Systematic recruitment, every 3rd name at 2 sites: a children’s support organization (case) and a mixed boarding school (control)	*N* = 153 Formerly abducted *N* = 82 Non-abducted *N* = 71	War affected adolescents Boys mean age = 15.5 years Girls mean age = 15.2 years Cases: 64% male; 36% female Controls: 61% male; 39% female 100% of controls in secondary school, 12.2% of cases in secondary school
Betancourt, Agnew-Blais et al., 2010	Sierra Leone	Prospective cohort Collected at baseline and time 2	Non random selection Two stage method: 1) master list of youth in care 2) Invited youth between ages 10–18 with contact information	*N* = 152 60% of *N* = 260 interviewed at both times	Former child soldiers Mean age = 17.4 years 89% male 11% female
Betancourt et al., 2011	Sierra Leone	Cross-sectional Partially nested in a longitudinal study	Non random selection Longitudinal participants from those who participated in one follow up visit, new participants recruited with NGO outreach lists	*N* = 273 *N* = 146 from longitudinal study and *N* = 127 newly recruited for study (50% male, 50% female)	Former child soldiers Mean age = 16.55 (SD 2.61) 71% male 29% female
Betancourt, Borisova et al., 2010	Sierra Leone	Prospective cohort Collected at baseline and time 2, approximately 2 years later	Non random selection Two stage method: 1) master list of youth in care 2) Youth aged 10–18 who did not have a severe disability participated	*N* = 156 60% of *N* = 260 interviewed at both times	Former child soldiers Mean age = 15.13 years 88% male 12% female
Betancourt, Brennan et al., 2010	Sierra Leone	Prospective cohort Collected at baseline (2002), time 2 (2004), and time 3 (2008)	Non random selection Sample from a master list of youth assisted by program. Youth aged 10–17 with contact information invited to participate.	*N* = 260 56.5% (*N* = 147) assessed at time 2 68.8% (*N* = 179) assessed at time 3	Former child soldiers Mean age at time 1 = 15.13 (SD = 2.22) 89% male 11% female
Johnson et al., 2008	Liberia	Cross-sectional	Random selection Population based multi stage random cluster of households	*N* = 1666 98.2% of *N* = 1696 attempted interviews	Adults in Liberia; 1/3 were former combatants Mean age = 41 years 47.2% male 52.8% female
Johnson et al., 2010	Democratic Republic of Congo	Cross-sectional	Non random selection Accessible population based cluster (some originally selected villages were inaccessible due to weather and security concerns)	*N* = 998 98.9% of *N* = 1005 households surveyed	Adults in conflict-affected provinces and districts Mean age = 40.1 years 40.6% male 59.4% female
Johnson et al., 2014	Kenya	Cross-sectional	Random sampling Systematic sampling of 90 villages and 10 households to assess election-related violence	*N* = 916 95.8% of *N* = 956 households samples	Adults in Kenya Mean age = 37.3 years 40% male 60% female
Cardozo et al., 2000	Kosovo	Cross-sectional	Random selection Two-stage cluster sampling	*N* = 1358 Only women included in relevant analysis, *N* = 825	Kosovar ethnic Albanians aged 15+ years 45.3% 15–34 34.1% 35–54 10.9% 55–64 9.7% 65+ 37.7% male 62.3% female
Sabin et al., 2003	Guatemalan refugees living in Mexico	Cross-sectional	Non random selection Convenience sample of 5 camps; all households sampled in 4 camps, every 3rd house in 1 camp	*N* = 170 93% of *N* = 183 households	Adults and children in Mayan refugee camps Mean age = 37.9 years 42% male 58% female
Wolfe et al., 1998	USA	Retrospective cohort Nested in longitudinal study. Baseline within 5 days of return from deployment, time 2 18–24 months later.	Non random selection Included women who completed the mailed sexual harassment questionnaire	*N* = 160 66.7% of *N* = 240 women assessed at baseline	Returned veterans of the Persian Gulf War Mean age = 28.2 years (SD = 6.8) 100% female
Washington et al., 2013	USA	Cross-sectional Pertinent result presented as case (PTSD) control (no PTSD)	Random selection Population-based stratified sample Included those who completed the PTSD screener	*N* = 3598 99.6% of *N* = 3611	Veterans who had been called to duty Mean age = 46.8 (SD = 17.3) for PTSD positive and 57.4 (SD = 17.0) for PTSD negative women 100% female
Kang et al., 2005	USA	Case control Nested data from a population based survey Cases: PTSD Controls: did not meet criteria for PTSD	Random selection Stratified sample to include each subgroup of military personnel	*N* = 11,441 76.3% of *N* = 15,000 sampled	Gulf War veterans Mean age: Females: with PTSD = 39.1; without PTSD = 38.1 Males: with PTSD 40.4; without PTSD 39.6 81.4% male 18.6% female
HIV acquisition/disease progression and mental health outcomes					
Epino et al., 2012	Rwanda	Cross-sectional From a prospective cohort	Non random selection Patients from clinics	*N* = 610	HIV-positive adults who initiated lifelong ART Mean age = 38 (SD = 10) 38% male 62% female Mean CD4 count =214 (SD = 92)
Mugisha, Muyinda, Wandiembe et al., 2015	Uganda	Cross-sectional Baseline data from a project delivering a kinship intervention for post-conflict mental health	Random selection Two-stage cluster sample stratified at the sub-county	*N* = 2361 98% with complete data of *N* = 2406	Adult residents of 3 of the most war affected districts 23.5% 18–24 years 27.3% 25–34 years 20.8% 35–44 years 28.5% 45–54 years 37.5% male 62.5% female
Mugisha, Muyinda, Malamba et al., 2015	Uganda	Cross-sectional Nested in project delivering a kinship intervention for post-conflict mental health	Random selection Multistage sampling for a representative sample from 3 districts	*N* = 2361 98% who had complete data included of *N* = 2406	Adult residents of 3 of the most war affected districts 23.8% 18–24 years 27.1% 25–34 years 20.7% 35–44 years 28.4% 45+ years 37.5% male 62.5% female
Muldoon et al., 2014	Uganda	Cross-sectional From a larger community-based study of sex workers	Non random selection Recruited through peer/sex worker led outreach in bars and hotels, and community-led outreach to former IDP camps	*N* = 129	Formerly abducted by the Lords Resistance Army Median age = 22 years (IQR:20–26) 100% female 96.1% from Acholi tribe
M.H. Cohen et al., 2009	Rwanda	Cross-sectional Baseline data from a prospective cohort study	Non random selection Mainly recruited by Rwandan women’s associations	*N* = 850 91% of *N* = 936 with available mental health data	HIV-positive and HIV-negative women About half of each group experienced genocidal rape Mean age = 36.4 100% female
M.H. Cohen et al., 2011	Rwanda	Prospective cohort Baseline, 6, 12, and 18 months later	Non random selection Recruited from Rwandan women’s associations and HIV clinics in Kigali	*N* = 698 74.6% of *N* = 936 who completed baseline HTQ and at least 1 post-baseline HTQ	HIV-positive and HIV-negative women 50% of each group experienced genocidal rape Mean age = 36.7 (SD = 8.3) 100% female
Other associations between mental health and HIV acquisition and disease progression					
Gard et al., 2013	Rwanda	Cross-sectional Baseline data nested in a prospective cohort study	Non random selection Recruited Rwandan women’s associations and clinical sites for HIV patients	*N* = 922 98.5% of *N* = 936 women who completed the Health-Related Quality of Life measure	HIV-positive and HIV-negative women 50% of each group experienced genocidal rape 20.8% under 30 years 48.4% aged 30–40 years 30.8% over 40 years 100% female
Kohli et al., 2014	Democratic Republic of Congo	Cross-sectional Baseline data from a randomized community trial	Non random selection Included if provided family rejection information and had experienced at least 1 traumatic event in the past 10 years	*N* = 315	Conflict-affected adult women 1.9% 16–19 years 14.6% 20–24 years 28.25% 25–34 years 22.54% 35–44 years 29.52% 45–60 years 3.17% over 60 years 100% female
Sinayobye et al., 2015	Rwanda	Cross-sectional Baseline data from 2005 RWISA prospective cohort	Non random selection HIV+ women, approximately 50% of whom experienced rape during the genocide	*N* = 710	HIV+ women over age 15, ART naïve Mean age = 34.9 ± 7.0 100% female

*ART* Antiretroviral therapy, *HIV* Human Immunodeficiency Virus, *HTQ* Harvard trauma questionnaire, *IDP* Internally displaced person, *PTSD* Post traumatic stress disorder, *USA* United States of America

**Table 2:** Study outcomes for association between mental health and HIV risk

First author & Year	Mental health disorders	Mental health scales	HIV risk measures	Results
Mental health and HIV serostatus/HIV-related outcomes				
Adedimeji et al., 2015 Rwanda	Depression PTSD	Center for Epidemiologic Studies Depression Scale (CES-D) Harvard Trauma Questionnaire (HTQ)	HIV serostatus Had sex last 6 months Condom use at least 50% of time last 6 months History of ever exchanging sex for cash or help History of a non-HIV STI	Depression (*p* < 0.001) but not PTSD (*p* = 0.06) was related to HIV serostatus Depression (*p* = 0.002) but not PTSD (*p* = 0.09) was related to sex in the last 6 months; women who had sex did not have different odds of depression scores between 16 and 26 (OR = 0.88, CI 0.64, 1.22) but had decreased odds of depression scores 27+ (OR = 0.57, CI 0.04, 0.81) and no different odds of symptomatic PTSD (OR = 0.78, CI 0.60, 1.03) Depression (*p* = 0.04) and PTSD (*p* = 0.006) were related to 50% condom use in the last 6 months; women who used condoms had greater odds of depression scores between 16 and 26 (OR = 1.84, CI 1.20, 2.82) but not scores 27+ (OR = 1.36, CI 0.87, 2.54) and decreased odds of symptomatic PTSD (OR = 0.60, CI 0.42, 0.86) Depression (*p* = 0.02) and PTSD (*p* = 0.003) were related to exchange sex; women who had exchanged sex had greater odds of depression scores between 16 and 26 (OR = 1.82, CI 1.19, 2.77) and 27+ (OR = 1.74, CI 1.10, 2.76) and greater odds of being symptomatic for PTSD (OR = 1.68, CI 1.19, 2.36) Depression (*p* = 0.04) but not PTSD (*p* = 0.74) related to history of a non-HIV STI; women with a non-HIV STI had greater odds of depression scores between 16 and 26 (OR = 2.02, CI 1.39, 3.09; AOR = 1.64, CI 1.01, 2.65) but not depression scores of 27+ (OR = 1.50, CI 0.94, 2.41; AOR = 1.11, CI 0.65, 1.89) nor symptomatic PTSD (OR = 1.07, CI 0.77, 1.50)
Adler et al., 2011 USA	PTSD	PTSD Checklist (PCL)	Risked STD by having unprotected sex	PTSD at time 1 predicted sex without a condom four months later (OR = 1.57, CI 1.20, 2.04)
Kinyanda et al., 2012 Uganda	Depression	Hopkins Symptom Checklist (HSCL-15)	High risk sexual behaviors: sex outside marriage; sex in exchange for gifts; sex in exchange for money; sex in exchange for protection; sex with an older person; sex with someone known for less than a day; sex with uniformed personnel; sex with more than one partner	High-risk sexual behavior was marginally related to MDD amongst males in univariate analysis (OR = 1.61, 95% CI 0.99–2.62, *p* = 0.06) but not females (OR = 1.17, 95% CI 0.68–2.01, *p* = 0.57). High-risk sexual behavior was related to MDD amongst males (OR = 1.70, 95% CI 1.01–2.86, *p* = 0.05) in multivariable analysis but not females (OR = 1.03, 95% CI 0.59–1.80, *p* = 0.91).
Kinyanda et al., 2016 Uganda	Depression	HSCL-25	Sexual intimate partner violence (IPV) (‘force you to have sex when you don’t want to’)	Females who experienced sexual IPV had greater odds of probable MDD (AOR = 4.20, CI 1.54, 11.46)
Malamba et al., 2016 Uganda	Depression PTSD	HSCL-25 HTQ	HIV serostatus	Those with MDD symptoms had greater odd of testing positive for HIV (UOR = 2.70, CI 1.95, 3.75; AOR = 1.89, CI 1.28, 2.80) Those with PTSD symptoms had greater odds of testing positive for HIV (UOR = 1.90, CI 1.30, 2.78; AOR = 1.44, CI 1.06, 1.96)
Svetlicky et al., 2010 Lebanon	PTSD	PTSD Inventory	Risky sexual activities (3 items including sex without protection against sexually transmitted diseases)	No relationship was found between PTSD and risky sexual activities^a^
Talbot et al., 2013 Rwanda	PTSD	PCL	Laboratory STI testing HIV risk taking behavior: Exchanging sex for drugs, money, or favors; Having sex with an HIV-infected or status unknown partner; Having two or more sexual partners within the past 3 months	Rates of STI were too low to evaluate associations with PTSD make any conclusions. Higher PTSD symptoms correlated with increased HIV risk-taking behavior (*r* = 0.24, *p* = 0.006) at baseline. PTSD symptoms were related to baseline HIV risk (0.01, *p* = 0.002) in a growth model; for each 1 point increase of trauma symptoms there was a 0.01 unit increase in baseline HIV risk
B.E. Cohen et al., 2012 USA	Depression PTSD Comorbid depression and PTSD	ICD-9-CM diagnostic codes	Sexually transmitted infections: cervical dysplasia; genital herpes; genital warts; chlamydia; gonorrhea; trichomonas; and other STIs	All STIs except chlamydia were associated with PTSD. Cervical dysplasia AOR = 1.86 (CI 1.61–2.16), Genital herpes AOR = 1.69 (CI 1.36–2.08), Genital warts AOR = 1.83 (CI 1.45–2.31), Chlamydia AOR = 1.66 (CI 0.93–2.96), Gonorrhea AOR = 3.12 (CI 1.51–6.44), Trichomonas AOR = 1.60 (CI 1.08–2.39), Other STIs AOR = 1.83 (CI 1.52–2.21) All STIs were associated with depression. Cervical dysplasia AOR = 2.35 (CI 2.12–2.59), Genital herpes AOR = 2.51 (CI 2.20–2.87), Genital warts AOR = 2.44 (CI 2.09–2.86), Chlamydia AOR = 2.21 (CI 1.49–3.27), Gonorrhea AOR = 3.99 (CI 2.38–6.71), Trichomonas AOR = 2.38 (CI 1.85–3.06), Other STIs AOR = 2.21 (CI 1.95–2.53) All STIs were most strongly associated with comorbid PTSD and depression. Cervical dysplasia AOR = 2.65 (CI 2.41–2.91), Genital herpes AOR = 2.55 (CI 2.24–2.91), Genital warts AOR = 2.97 (CI 2.56–3.43), Chlamydia AOR = 2.58 (CI 1.80–3.70), Gonorrhea AOR = 4.74 (CI 2.91–7.71), Trichomonas AOR = 3.75 (CI 3.01–4.66), Other STIs AOR = 2.92 (CI 2.59–3.28)
Sexual violence and mental health outcomes				
Amone-P’olak et al., 2013 Uganda	Depression and anxiety	Acholi Psychosocial Assessment Instrument (APAI)	Sexual abuse measured by one item in the War Trauma Screening scale	Sexual abuse (β = 0.32, SE = 0.16, *p* < 0.001) predicted symptoms of depression and anxiety for female but not male youths in multivariate analysis.
Roberts et al., 2008 Uganda	PTSD	HTQ	Rape or sexual abuse	Those who reported rape or sexual abuse had greater odds of PTSD symptoms (AOR = 1.76, CI 1.01, 2.75) but not depression symptoms (NR)
Nakimuli-Mpungu et al., 2013 Uganda	Depression PTSD	Self- reporting questionnaire (SRQ-20) HTQ	Experienced sexual violence *HIV serostatus*	Experiencing sexual violence was significantly related to PTSD symptom scores (β = 3.75, SE = 1.01, *p* < 0.05) but not depression symptom scores (β = 0.54, SE = 0.45). Being HIV-positive was not significantly related to depression (β = 0.51, SE = 0.43) or PTSD (β = −1.41, SE = 0.94) scores.
Okello et al., 2007 Uganda	Depression Anxiety PTSD	MINI-KID	Sexual torture (undefined) Being forced to marry	Quantitative results not presented in a table, but the stated that no trauma event (including sexual torture and being forced to marry) showed any significant relationship with any diagnosis of PTSD, major depression and generalized anxiety disorder.^a^
Betancourt, Agnew-Blais, et al., 2010 Sierra Leone	Depression and anxiety	A measure developed by the Oxford Refugee Studies Program for use among former child soldiers includes a subscale for anxiety, depression, and hostility	Rape as part of Child War Trauma Questionnaire	Surviving rape predicted an increase in depression over time (*b* = 2.58, *p* = 0.01) after controlling for demographic and war-related experiences. When perceived discrimination was included, the strength of the relationship between rape and depression is reduced, (*b* = 1.65, *p* = 0.08). When protective factors were added, there was no longer a relationship between rape and depression. Surviving rape was significantly associated with higher levels of anxiety (*b* = 5.35, *p* < 0.001) even after perceived discrimination and protective factors were controlled for.
Betancourt et al., 2011 Sierra Leone	Depression and anxiety	HSCL-25	Rape as part of Child War Trauma Questionnaire	No significant relationship between rape and depression after controlling for multiple variables *b* = 2.42 (CI -0.99, 5.84). Rape was significantly related to anxiety *b* = 2.85 (CI 0.45, 5.26, *p* = 0.05). A smaller percentage of boys experienced rape (5%) compared to girls (44%), but the effect of rape on anxiety was significant among male child soldiers and not for females (*b* = −6.42, *p* = 0.05).
Betancourt, Borisova, et al., 2010 Sierra Leone	Depression and anxiety	Oxford Refugee Studies Program measure for use among former child soldiers	Rape as part of Child War Trauma Questionnaire	Rape was correlated to depression symptoms (*r* = 0.24, *p* ≤ 0.01) and anxiety symptoms (*r* = 0.38, *p* ≤ 0.001). Rape was not predictive of depression at T2, adjusting for all covariates (*b* = 1.74, CI -0.53, 4.00). Rape was the strongest predictor of anxiety at T2 controlling for anxiety levels at T1 (*b* = 4.06, CI 1.49, 6.62, *p* < 0.05) and adjusting for all other covariates.
Betancourt, Brennan et al., 2010 Sierra Leone	Depression and anxiety	Oxford Refugee Studies Program measure for use among former child soldiers	Rape as part of Child War Trauma Questionnaire	Rape was associated with higher baseline levels of internalizing problems (depression/anxiety) (*b* = 4.60, *p* < 0.05). After adjusting for all hardship and protective factors, among time-invariant predictors, only being raped remained significantly related to depression/ anxiety (*b* = 4.34, *p* = 0.039).
Johnson et al., 2008 Liberia	Depression PTSD	Patient Health Questionnaire 9 PTSD Symptom Scale Interview (1 month recall)	Sexual violence defined as any violence, physical or psychological, carried out through sexual means or by targeting sexuality and included rape and attempted rape, molestation, sexual slavery, being forced to undress or being stripped of clothing, forced marriage, and insertion of foreign objects into the genital opening or anus, forcing 2 individuals to perform sexual acts on one another or harm one another in a sexual manner, or mutilating a person’s genitals.	Adults who experienced sexual violence were more likely to meet criteria for PTSD (69% vs. 38%, *p* < 0.001) and MDD (57% vs. 37%, *p* = 0.002) compared to adults who did not experience sexual violence. The weighted prevalence of PTSD (81% vs. 46%, *p* < 0.001) and MDD (64% vs.42%, *p* = 0.003) was higher among male former combatants who had experienced sexual violence compared to those who had not. The weighted prevalence of PTSD (74% vs. 44%, *p* = 0.005) was higher but not MDD (63% vs.55%, *p* = 0.51) among female former combatants who experienced sexual violence compared to those who had not. Noncombatant sexual violence was not related to MDD (32% vs. 29%, *p* = 0.73) nor PTSD (39% vs. 36%, *p* = 0.74) for men nor MDD (48% vs. 36%, *p* = 0.15) nor PTSD (56% vs.36%, *p* = 0.09) for women. Those who experienced lifetime sexual violence had 1.39 (*p* = 0.04) the odds of MDD and 2.67 (*p* < 0.001) the odds of PTSD compared to those who did not experience sexual violence.
Johnson et al., 2010 Democratic Republic of Congo	Depression PTSD	Patient Health Questionnaire–9 PTSD Symptom Scale Interview (PSS-I)	Sexual violence – defined above	The prevalence of MDD was significantly higher for those who experienced sexual violence (60.4%) compared to those who did not experience sexual violence (30.7%, *p* < 0.001); The prevalence of PTSD was significantly higher for those who experienced sexual violence (70.2%) than those who did not experience sexual violence (40.3%, *p* < 0.001) . The prevalence of MDD for females who experienced conflict-related sexual violence was significantly higher (67.7%) than for those who did not experience conflict-related sexual violence (30.3, *p* < 0.001). The prevalence of PTSD for females who experienced conflict-related sexual violence was significantly higher (75.9%) than for those who did not experience conflict-related sexual violence (44.4%, *p* < 0.001). There were no differences in the prevalence of MDD (47.5% vs. 36.3%, *p* = 0.18) or PTSD (56% vs. 41.7%, *p* = 0.17) for men who did and did not experience conflict-related sexual violence. There were no differences in the prevalence of MDD (50.7% vs. 38.4%, *p* = 0.38) or PTSD (61.5% vs. 44.1%, *p* = 0.34) for men nor of MDD (72.9% vs. 40.1%, *p* = 0.07) or PTSD (83.6% vs. 52.4%, *p* = 0.06) for women who experienced community based sexual violence.
Johnson et al., 2014 Kenya	Depression PTSD	Patient Health Questionnaire–9 PSS-I	Sexual violence – defined above	31% of those who experienced sexual violence had anxiety and depression before the 2007 election, 45% who experienced sexual violence had anxiety and depression during the election, and 33.7% who experienced sexual violence had anxiety and depression after the 2007 election. The weighted prevalence of MDD (41.0%, CI 27, 55 vs. 35.0%, CI 29.2, 40.8) and PTSD (40.1%, CI 28.6, 51.6 vs. 30.9%, CI 25, 36.8) were not significantly different between those who reported sexual violence and those who did not report sexual violence.
Cardozo et al., 2000 Kosovo	PTSD	HTQ	Rape	Rape was not related to PTSD symptoms: 21.6% or women who reported rape had symptoms of PTSD vs. 16.92% of women who did not report rape, *p* = 0.49; AOR = 1.68, CI 0.69, 4.08
Sabin et al., 2003 Guatemalan refugees living in Mexico	Depression Anxiety PTSD	HSCL-25 HTQ	Sexual abuse or rape reported as traumatic event	Sexual abuse or rape was independently associated with anxiety (*p* = 0.02) but sexual abuse did not remain significant in the full model. All rape survivors (*N* = 6, 100%) experienced anxiety. Sexual abuse or rape was not related to PTSD or depression.^a^
Wolfe et al., 1998 USA	PTSD	Mississippi Scale for Combat-related PTSD	Sexual assault defined as a sexual experience that was unwanted and involved the use or threat of force (attempted or completed rape) either by strangers or people you knew	Women who were sexually assaulted experienced a significant 18.9 point increase in PTSD scores (M = 91.83, SD = 22.69) compared to women with no sexual harassment (M = 71.36, SD = 17.53). Women who were sexually assaulted had increased risk for PTSD compared to women who were only physically (12.5 point difference) or verbally (15.9 point difference) harassed.
Washington et al., 2013 USA	PTSD	7-item screen for DSM IV PTSD	History of military sexual assault	Women with PTSD were significantly more likely to have had experienced sexual assault in military (43% vs. 5.1%, *p* < 0.001).
Kang et al., 2005 USA	PTSD	PCL	Sexual assault	Among female (AOR = 5.41; 95% CI 3.19, 9.17) and male (AOR = 6.21 CI 2.26, 17.04) veterans, sexual assault was significantly associated with PTSD even while controlling for other covariates.
HIV acquisition/disease progression and mental health outcomes				
Epino et al., 2012 Rwanda	Depression	HSCL-15	CD4 count	There was not a significant difference in depression for those with <=200 CD4 cell count (25.5) and > 200 CD4 count (26) (*p* = 0.58).
Mugisha, Muyinda, Wandiembe et al., 2015 Uganda	PTSD	Mini-International Neuropsychiatric Interview (MINI)	HIV status *Sexual trauma events*	Those reporting HIV+ status had greater odds of having PTSD (UOR = 2.09, CI 1.48, 2.95) Those who experienced 1–2 sexual trauma events had greater odds of having PTSD in the unadjusted (UOR = 2.6, CI = 1.63, 4.15) but not the adjusted (AOR = 1.23, CI 0.73, 2.07) model Those who experienced 3+ sexual trauma events had greater odds of having PTSD (UOR = 5.65, CI 3.33, 9.61; AOR = 2.02, CI 1.08, 3.76)
Mugisha, Muyinda, Malamba et al., 2015 Uganda	Depression	MINI	HIV status High risk sexual behaviors Receiving HIV treatment	HIV+ status was related to MDD (UOR = 2.85, CI 2.04, 3.96), after adjusting for sex and age (AOR = 2.63, CI 1.87, 3.70), and in the multivariate model (OR = 1.83, CI 1.22, 2.74) High risk sexual behavior was not related to MDD in the unadjusted (UOR = 1.13, CI 0.77, 1.67) or adjusted model (AOR = 1.37, CI 0.91, 2.09) Receiving HIV treatment was related to MDD in the adjusted model (AOR = 3.22, CI 1.08, 9.57) but not the unadjusted model (UOR = 2.03, CI 0.85, 4.85)
Muldoon et al., 2014 Uganda	Depression and anxiety	APAI	All participants had exchanged sex for money or resources in the previous 30 days	For all participants the mean score for the depression sub-scale was 12.84 (SD = 4.79) and the mean score for the anxiety sub-scale was 8.76 (SD = 5.14). No cut off score is defined for symptomatic for either subscale.
M.H. Cohen et al., 2009 Rwanda	Depression PTSD	CES-D HTQ	About 50% of participants in each group of HIV-positive and HIV-negative experienced genocidal rape CD4 cell counts	Women with HIV infection were more likely than HIV-negative women to have clinically significant depression (81% vs. 65%, *p* < 0.0001) and MDD (31% vs. 23%, *p* < 0.047). Women with more advanced HIV, indicated by CD4 cell counts < 200 = mL (OR 4.97, CI 2.93, 8.45), were the most likely to have depressive symptoms. Women who had experienced genocidal rape were more likely to have PTSD in unadjusted analyses (OR = 1.63, CI 1.23, 2.15). Depressive symptoms were higher in women who had a history of genocidal rape (OR = 1.56, CI 1.12, 2.16).
M.H. Cohen et al., 2011 Rwanda	Depression PTSD	CES-D HTQ	About 50% of participants in each group of HIV-positive and HIV-negative experienced genocidal rape	HIV-positive status was related to increased symptoms of depression (81.5% vs. 63.8%, p < 0.0001), marginally related to symptoms of PTSD (59.6 vs. 67.5%, *p* = 0.081), and not related to MDD (29.2% vs. 22.7%, *p* = 0.11) compared to HIV-negative status at baseline. There was a continued reduction in PTSD at each follow-up visit for both HIV-positive and HIV-negative groups (6 month change = −0.78, *p* < 0.0001; 12 month change = − 0.9, *p* < 0.0001; 18 month change = − 0.84, *p* < 0.0001). HIV-positive status (b = 0.03, *p* = 0.38) was not related to PTSD improvement from baseline to 18-month follow up. All participants had fewer depressive symptoms at 18 months follow up compared to baseline (77% vs. 57%). In changes from baseline to visit 4, experiencing genocidal rape was significantly associated with reduced PTSD.^a^
Other associations between mental health and HIV acquisition and disease progression				
Gard et al., 2013 Rwanda	Depression PTSD	CES-D HTQ	About 50% of participants in each group of HIV-positive and HIV-negative experienced genocidal rape	HIV-positive women had higher depression scores than HIV-negative participants (23.67, SD = 9.19 vs. 20.79, SD = 9.60, *p* < 0.001). More HIV-positive women met criteria for depression than HIV-negative women (81.46% vs.64.58%, *p* < 0.001). There was no difference in PTSD scores between HIV-positive and HIV-negative women (2.31, SD = 0.69 vs. 2.4 SD = 0.67, *p* = 0.09). A greater percentage of HIV-negative compared to HIV-positive women experienced elevated PTSD scores (65.63% vs. 57.8%, *p* = 0.05).
Kohli et al., 2014 Democratic Republic of Congo	Depression PTSD	HSCL-15 HTQ	Rape	Rape or sexual assault in the past 10 years was related to increased symptoms of PTSD (β = 0.35, *p* < 0.001) and depression (β = 0.29, *p* < 0.001) in multivariate regression.
Sinayobye et al., 2015 Rwanda	Depression PTSD	CES-D HTQ	CD4 count	Depression scores were associated with CD4 count (*p* < 0.001) with: CD4 counts > 350 having a mean depression score of 22.4 ± 9.3; CD4 count 200–350 having a mean depression score of 23.0 ± 8.2; CD4 count < 200 having a mean depression score of 25.8 ± 9.1 PTSD scores were not associated with CD4 count (*p* = 0.60) with: CD4 counts > 350 having a median (IQR) PTSD score of 2.1 (1.7–2.7) CD4 count 200–350 having a median (IQR) PTSD score of 2.1 (1.8–2.8) CD4 count < 200 having a median (IQR) PTSD score of 2.2 (1.8–2.8)

*AOR* adjusted odds ratio, *ART* Antiretroviral therapy, *CI* confidence interval, *MDD* major depressive disorder, *NR* not reported, *OR* odds ratio, *SE* Standard Error, *SD* standard deviation

^a^Effect size data are reported where available; textual descriptions of results are reported when that was all that the authors present

Table 1 provides information on location, study design, sampling strategy, study size, and participant characteristics for each included article. Although most studies were cross-sectional, many articles presented the expected directionality of the relationship based on which variable was considered the exposure and which was considered the outcome. Results are organized by the outcome variable reported by the authors (using the authors’ expected directionality of the association) as follows: mental health and HIV serostatus/HIV-related outcomes; sexual violence and mental health outcomes; HIV acquisition/disease progression and mental health outcomes; and other associations. Under other associations, we include studies that met the inclusion criteria but that did not specify the expected directionality of the associations. Twenty-five studies were from sub-Saharan Africa, five were from the United States of America (USA), and one each was from the Middle East, Europe, and Latin America. Most studies were cross-sectional, though three time-series studies and seven cohort studies reported longitudinal data. Most studies had non-random sampling of participants, though 14 studies utilized a form of random sampling. Overall, 110,818 participants were included across articles. However, there was overlap in participants across four papers discussing child soldiers in Sierra Leone [22–25], in five papers discussing HIV-positive and negative women, half of whom survived rape during the genocide in Rwanda [26–30], in two papers from Uganda [31, 32], and in two papers from the Wayo-Nero Study in Uganda [33, 34]. The smallest study included 120 participants and the largest study included 71,504 participants.

Table 2 presents each of the included studies by author and year, country, mental health disorders and measurement scales, HIV risk measures, and the relationship found between mental health and HIV-related outcomes. Most studies reported mental health outcomes either in relation to sexual violence or other factors related to HIV acquisition and disease progression. Eight studies reported HIV serostatus or HIV risk outcomes. Three studies reported other outcomes but included analyses of the association between mental health and HIV acquisition and disease progression. In several studies, the relationship between HIV-related factors with mental health was not the main objective, but rather one of many results reported.

### Mental health and HIV serostatus/HIV-related outcomes

Of the eight studies that reported HIV serostatus or HIV-related outcomes, five included HIV sexual risk behaviors, two reported HIV serostatus, two reported other STIs, and one reported intimate partner sexual violence.

Four HIV sexual risk behavior studies found some relationship between mental health and HIV risk behaviors. A study from Uganda found a relationship between depression and at least one of eight sexual risk behaviors amongst males in multivariate analysis in a war-affected community (odds ratio *(OR)* = 1.70, *p* = 0.05) [32]. In one Rwandan study, increased symptoms of PTSD were correlated with at least one of three HIV risk-taking behaviors (*r* = 0.24, *p* = 0.0006) [35]. In another Rwandan study, women who had exchanged sex had greater odds of depression (*OR* = 1.74, confidence interval (CI) 1.10, 2.76) and PTSD (*OR* = 1.68, CI 1.19, 2.36) [30]. Women who used condoms at least 50% of the time had decreased odds of PTSD (OR = 0.60, CI 0.42, 0.86), but increased odds of elevated depression scores (*OR* = 1.84, CI 1.20, 2.82) [30]. Women who had sex in the last six months had decreased odds of depression (*OR* = 0.57, CI 0.04, 0.81) [30]. A longitudinal study from the USA showed that PTSD predicted unprotected sex four months later (OR = 1.57, CI 1.20, 2.04) [36]. In Lebanon, no relationship was found between PTSD and risky sexual activities [37].

Two studies focused on HIV serostatus as an outcome. Depression (*p* < 0.001) but not PTSD (*p* = 0.06) was related to HIV positive serostatus among female survivors of the Rwandan genocide in a cross-sectional, non-randomly selected cohort [30]. In a randomly selected cross-sectional study in Uganda, men and women with depression (adjusted odds ratio *(AOR)* = 1.89, CI 1.28, 2.80) and PTSD (*AOR* = 1.44, CI 1.06, 1.96) had greater odds of testing positive for HIV [38].

Two studies reported STIs other than HIV as an outcome. Female survivors of the Rwandan genocide who had a non-HIV STI had greater odds of elevated depression scores (*AOR* = 1.64, CI 1.01, 2.65), but not PTSD (*OR* = 1.07, CI 0.77, 1.50) [30]. Female USA veterans of conflict in Iraq and Afghanistan who had PTSD were significantly more likely to have six of seven different STIs compared to veterans without any mental health diagnosis; female veterans with depression were more likely to have all seven STIs compared to those without any mental health diagnosis [39]. Those with comorbid depression and PTSD were even more likely to have all seven STIs compared to those without any mental health diagnosis with adjusted OR between 2.55 and 4.74. This study had the largest population amongst the included articles (*N* = 71,504) and represented the entire population of female veterans who met inclusion criteria.

Finally, one study from Uganda reported intimate partner sexual violence as an outcome. In this a cross-sectional study, females who experienced sexual violence from an intimate partner had greater odds of probable depression (*AOR* = 4.20, CI 1.54, 11.46) [31].

Overall, studies reported strong associations between depression and PTSD and HIV serostatus/HIV-related outcomes in African and American conflict-affected populations.

### Sexual violence and mental health outcomes

Twenty-two studies reported mental health outcomes: two reported depression only; six PTSD only; six depression and anxiety; six depression and PTSD; and two depression, anxiety, and PTSD. A large number of studies in this review (*n* = 16) were conducted in sub-Saharan Africa and reported the relationship between mental health and sexual violence, rape, or sexual abuse (as an HIV-related measure out of the victims’ control). The remaining studies in this category were conducted in the USA (*n* = 3), with Kosovar ethnic Albanians (*n* = 1), and with Guatemalan refugees in Mexico (*n* = 1).

Most studies found a positive association between sexual violence and poor mental health; three studies found no association. Sexual abuse predicted depression and anxiety for female Ugandan youth (*β* = 0.32, *p* < 0.001) [40]. Experiencing sexual violence was related to PTSD in a study among Ugandan adults (β = 3.75, *p* < 0.05) [41]. Similarly, in another study of Ugandan adults, those who experienced three or more sexual trauma events had greater odds of PTSD (*AOR* = 2.02, CI 1.08, 3.67) [34]. For Ugandan adults living in camps for internally displaced people, those who reported rape had greater odds of PTSD (*AOR* = 1.76, CI 1.01, 2.75), but not depression [42]. Amongst adult and adolescent Guatemalan refugees in Mexico, sexual abuse was independently associated with anxiety, but did not remain significant after controlling for other variables [43]. Among Kosovar ethnic Albanian women, rape was not related to PTSD (*AOR* = 1.68, CI 0.69, 4.08) [44]. It is notable that in this study, rape was only identified in 4.4% of women (*N* = 60), [44].

Three studies by the same study team examined the relationship of sexual violence with depression and PTSD in different countries. The studies utilized different methodologies and had different outcomes. Sexual violence was related to PTSD and depression in Liberian adults, with prevalence of PTSD higher for male (81% vs. 46%, *p* < 0.001) and female (74% vs. 44%, *p* = 0.005) former combatants who experienced sexual violence; depression was higher for male former combatants who experienced sexual violence compared to those who did not (64% vs.42%, *p* = 0.003) [45]. In the Democratic Republic of Congo, prevalence of PTSD (70.2% vs. 40.3%, *p* < 0.001) and depression (60.4% vs. 30.7%, *p* < 0.001) were higher for participants who experienced sexual violence compared to those who did not; prevalence of PTSD and depression were higher for females who experienced conflict-related sexual violence compared to females who did not, respectively (75.9% vs. 44.4%, *p* < 0.001 and 67.7% vs. 30.3%, *p* < 0.001) [46]. During the 2007 election violence in Kenya, the prevalence of sexual violence was not significantly related to major depressive disorder or PTSD [47].

The Liberian study had the strongest methodological quality (utilizing a population based multistage random household cluster survey) and the study in the Democratic Republic of Congo had the weakest quality of the three (non random accessible population cluster). Although sexual violence in Kenya was not significantly related to major depressive disorder and PTSD, sexual violence was significantly related to suicidal ideation and suicide attempt [47].

Sexual torture and being forced to marry were not related to depression, anxiety, and PTSD amongst formerly war abducted adolescents in Uganda [48]. However, the study may not have had the statistical power to detect significance for such analysis as the primary objective was to compare formerly abducted and non-abducted adolescents. No trauma event had a significant relationship with mental health outcomes in this randomly selected population.

Four studies reported data from a non-randomly selected prospective cohort of child soldiers in Sierra Leone. In two studies with overlapping samples (one cross-sectional with 273 participants and one longitudinal with 156 participants), rape was significantly related to anxiety (*b* = 2.85, *p* = 0.05; *b* = 4.06, *p* < 0.05), especially in males, but not depression after controlling for other variables [23, 24]. In a slightly different sample, rape was associated with increased anxiety after controlling for discrimination and protective factors (*b* = 5.35, *p* < 0.001); rape significantly predicted an increase in depression over time, but this relationship lessened when perceived discrimination was added and was no longer significant when protective factors were added [22]. Rape was the only time-invariant predictor found to be related to depression/anxiety (considered together) after adjusting for hardship and protective factors (*b* = 4.34, *p* = 0.04) [25]. Combined, these studies demonstrate that rape (as a more extreme war event) is a strong predictor of anxiety over time amongst war-affected adolescents.

Three publications examined sexual assault and mental health in conflict-affected American military populations. Female veterans who returned from deployment in the Persian Gulf were more likely to experience PTSD if they had been sexually assaulted during their service compared to women who did not experience harassment, who only experienced verbal harassment, and who experienced physical harassment that was not sexual assault [49]. In cross-sectional analyses, female veterans with PTSD compared to those without PTSD were more likely to have experienced sexual assault while in the military (43% vs. 5.1%, *p* < 0.001) [50]. Sexual assault reports were significantly associated with PTSD for men (*OR* = 6.21, CI 2.26, 17.04) and women (*OR* = 5.41, CI 3.19, 9.17) after controlling for other variables [51]. Findings from these studies supported a relationship between sexual assault and poor mental health.

Overall, studies reported strong associations between sexual violence and all three mental health outcomes. Sexual violence was most strongly associated with PTSD [41, 42, 45, 46, 49–51], then depression [22, 24, 25, 40, 45, 46], and then anxiety mainly amongst male survivors [22–25, 40, 43].

### HIV acquisition/disease progression and mental health outcomes

Factors related to HIV acquisition and disease progression were associated with mental health outcomes in two studies that reported HIV sexual risk behavior, one that reported CD4 count, one that reported receiving HIV treatment, two that reported HIV status, and two that compared HIV-positive and HIV-negative participants and rape during genocide.

In Uganda, formerly conflict-abducted young women who recently exchanged sex for money or resources were assessed for mental health with a locally developed measure; mean depression and anxiety scores were 12.84 and 8.76, respectively [52]. Cut off scores defining symptomatic versus asymptomatic depression and anxiety were not provided in this study so no conclusion can be made regarding the association between HIV sexual risk with depression and anxiety. In another study in Uganda, adults with high risk sexual behavior did not have higher odds of depression (*AOR* = 1.37, CI 0.91, 2.09) [33].

Lower CD4 count indicates more advanced HIV disease progression and potentially increases transmissibility. In one non-randomly selected population in Rwanda, there was no difference in depression scores for adults with CD4 counts at or below 200 compared to adults with CD4 counts over 200 [53]. Receiving HIV treatment was associated with depression (*AOR* = 3.22, CI 1.08, 9.57) among a random selection of adults in Uganda [33]. Reporting an HIV-positive status was associated with both PTSD (*OR* = 2.09, CI 1.48, 2.95) [34] and depression (*OR* = 1.83, CI 1.22, 2.74) [33] among Ugandan adults.

In two publications from another non-randomly selected cohort in Rwanda, depression and PTSD in HIV-positive and negative women were examined both cross-sectionally and longitudinally. At baseline, HIV-positive women were more likely than HIV-negative women to have depression symptoms (81% vs. 65%, *p* < 0.0001) and HIV-positive women with CD4 counts below 200 were the most likely to have depression symptoms (*OR* = 4.97, CI 2.93, 8.45) [26]. Women who experienced rape during the genocide were more likely to have PTSD (OR = 1.63, CI 1.23, 2.15) and depression (*OR* = 1.56, CI 1.12, 2.16) in unadjusted analysis [26]. Over three follow-up visits at six, twelve, and eighteen months, HIV-positive and HIV-negative women had reduced PTSD scores; this improvement was not related to antiretroviral therapy (ART) use, but was related to HIV-positive status at follow-up three, and was related to rape during the genocide at follow-ups two and three [27]. Overall, studies examining mental health outcomes and factors related to HIV acquisition and disease progression reported both positive and null associations.

### Other associations between mental health and HIV acquisition and disease progression

Three studies used other outcomes for their primary analyses but provided data assessing the cross-sectional association between mental health and HIV acquisition and disease progression, making less clear the authors’ expected directionality of the relationship. One study in Rwanda (with some study population overlap with other included articles) examined quality of life amongst trauma-affected women with and without HIV. About half of the women in each group had experienced rape during the genocide. HIV-positive women experienced increased depression symptom scores compared to HIV-negative women (*p* < 0.001), but a greater percentage of HIV-negative women experienced elevated symptoms of PTSD [28]. In another study in Rwanda among HIV positive women (with study population overlap) depression (*p* < 0.001), but not PTSD (*p* = 0.60), was associated with CD4 count [29]. In the Democratic Republic of Congo, family rejection amongst survivors of sexual assault was more strongly related to depression, but increased PTSD was more strongly related to sexual assault [54]. Rape or sexual assault in the past ten years was associated with both increased PTSD (*β* = 0.35, *p* < 0.001) and depression (*β* = 0.29, *p* < 0.001).

Overall, studies in this category demonstrated inconsistent associations between mental health and HIV acquisition and disease progression.

## Discussion

Overall, the thirty-three studies included in this review suggest that symptoms of poor mental health are associated with increased sexual risk behaviors and HIV markers, and HIV exposures and HIV-related health status are associated with poor mental health symptoms in conflict-affected populations. Only five studies found no association between mental health and factors related to HIV acquisition and disease progression. Associations were strongest for mental health and HIV serostatus/HIV-related outcomes and sexual violence and mental health outcomes. Associations between HIV acquisition/disease progression and mental health outcomes and outcomes that examined other associations were more inconsistent. This could be partially attributed to the greater variety in the types of associations measured in these categories. For example, studies that examined sexual violence and mental health outcomes only included associations between sexual violence and mental health, given the large number of studies in this category. However, studies that examined HIV acquisition/disease progression and mental health outcomes looked at a variety of HIV-related correlates with mental health outcomes (e.g. CD4 count, sexual risk behaviors, HIV positive serostatus).

There were slight inconsistencies across studies in associations. For articles that found an association between one or more mental health disorders and factors related to HIV acquisition and disease progression, there were other articles, or findings within the same article, that failed to identify a relationship with one or more other disorders. These inconsistencies may represent real differences in various associations or populations. Inconsistencies in the findings may also be attributed to less rigor among some studies that did not find associations: non-random selection and small sample size [37], small numbers of relevant participants within the sample [43, 48], and low rates of the HIV-related variable [35]. Some studies that did not find associations had trends towards an association [47] or a marginal association [31]. Studies with strong rigor (large sample sizes of randomly selected participants or the entire population) found strong positive associations both in conflict-affected USA [39, 50, 51] and African populations [31, 38].

Our findings are similar to a review of HIV risk behaviors and trauma amongst migrants from low and middle-income countries [18]. Our study differs from the review by Michalopoulos et al. by including associations between mental health and factors related to HIV acquisition and disease progression in conflict-affected populations from high-income countries, focusing on measurable mental health disorders, and exclusively presenting quantitative relationships.

We only included mental health symptoms documented by validated scales to ensure that included studies met a minimal level of measurement rigor and to increase comparability of findings across studies. Most included articles meet the criteria for ‘protracted’ or ‘post-conflict’ [21, 47]. However, one study stands out as it specified reporting election-related violence rather than war or conflict; with 1133 deaths over 59 days, the setting met criteria for an ‘explosive’ event [21, 47]. This demonstrates the range of included conflict-affected settings. Because HIV disproportionally affects sub-Saharan Africa, much of the literature discussing conflict and HIV focuses on this geographic area. Similarly, a wide range of conflict-affected populations were included in this review, from soldiers fighting in their own country to returned American combat veterans, from orphans to child soldiers, from abducted civilians to those living in internally displaced persons camps. Unfortunately, there were not enough studies examining the same factors in the range of conflict-affected regions or populations to draw strong conclusions as to how these relationships might differ by region or population.

**Fig. 2:**
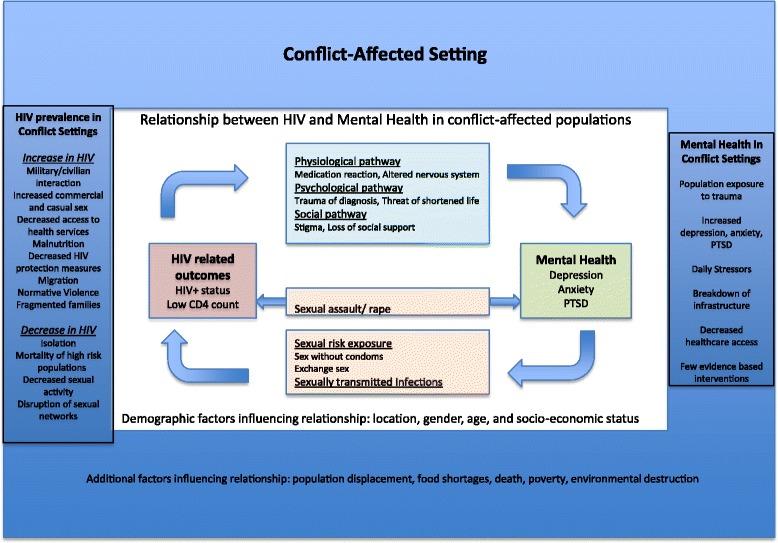
Relationship Between Mental Health & HIV in Conflict Settings. This figure illustrates a framework of the relationship between mental health and HIV serostatus and HIV-related outcomes among conflict-affected populations, based on this review and existing literature. The outside blue panel presents existing knowledge of factors in conflict-affected settings. HIV prevalence could increase or decrease in conflict-settings through various mechanisms [13]. Multiple factors contribute to increased risk for poor mental health in conflict-affected settings. Additional factors adversely affect conflict-affected populations. The inside box presents the relationship between mental health and HIV-related outcomes. HIV can physiologically, psychologically, and socially increase risk for mental health disorders. Health status (being HIV-positive and lower CD4 count) is associated with poor mental health. Poor mental health can influence HIV risk exposures (sexual risk behaviors and STIs). Surviving sexual assault is associated with poor mental health and HIV-related outcomes. Demographic factors can influence each relationship

We used findings from this review along with existing literature to develop a framework (Fig. 2). Mock et al. described how HIV prevalence could increase (via increased military and civilian interaction, increased commercial and casual sex, etc.) or decrease (via increased isolation, mortality of high-risk populations, etc.) in conflict-affected settings [13]. It is well established in the literature that conflict-affected populations are at risk for poor mental health [8, 12, 55], and often lack access to mental health services [56, 57]. Physiological, psychological, and social pathways can influence the relationship between HIV-related outcomes and mental health disorders [7]. What is lacking in the literature, to the best of our knowledge, is a framework that incorporates the relationship between mental health and factors associated with HIV acquisition and progression in conflict-affected populations.

Our findings provide evidence for this framework. Specifically, we identified studies that demonstrate health status (HIV-positive serostatus, lower CD4 count) is associated with increased depression [26–30, 38], that mental health disorders may influence HIV risk exposures (sexual risk behaviors [30, 32, 36] and STIs [30, 39]), and that surviving sexual assault may be associated with poor mental health [22–25, 40, 41, 43, 45, 46, 49–51].

There were several limitations to this review. First, only one reviewer identified, screened, and extracted data from included studies. Methodological rigor would be strengthened if two reviewers had independently completed each step and resolved any discrepancies. Although it was not possible to have two reviewers conduct all steps in the review process, a second, experienced reviewer was consulted when specific questions arose during the process and a third reviewer verified the extracted data. Second, the search terms for ‘conflict’ focused only on conflict and war. By excluding terms such as refugee, displaced persons, and asylum seekers we may have missed articles relevant to this review.

We examined only three mental health disorders, selected because they are common and frequently measured in conflict-affected populations. However, other disorders may be relevant in conflict-affected populations, specifically substance use, which has been shown to be common, harmful, and related to HIV transmission and risk in conflict-affected populations [58–60]. Finally, 23 of the 33 studies were cross-sectional, so the temporality of these relationships cannot be determined. No studies were identified in this review that examined the association between viral suppression and mental health; future studies should examine these associations. Longitudinal studies should also be conducted examining associations between mental health and factors related to HIV acquisition and disease progression. Future reviews could examine the associations between HIV acquisition or risk behaviors and substance use or other mental health disorders in conflict-affected populations.

There are several implications from this review. Considering the associations discussed in this paper, programs delivering HIV or other reproductive health services for conflict-affected populations should consider screening individuals who have HIV or STIs for mental health disorders using appropriate cross-culturally validated tools. Referrals could then be made, assuming availability of evidence-based interventions. Similarly, programs delivering mental health services should consider screening for HIV serostatus and associated risk factors. Since health infrastructure is often limited in conflict-affected settings, combining screening and services offers the potential to more systematically and holistically treat vulnerable individuals. An example where this has occurred is in Uganda, where an organization that provided mental health interventions for conflict survivors included HIV screening, referrals, and services to meet the unique mental health needs of people living with HIV [61].

At the policy level, by recognizing the relationships between mental health and factors related to HIV acquisition and disease progression in conflict settings, infrastructure can be integrated to offer mental health and HIV-related services simultaneously. Policy could also require monitoring of results to recognize any differences in separate treatment compared to integrated treatment of mental health and HIV-related services. Future research should employ stronger methods where possible – specifically, random selection of participants to decrease bias and longitudinal studies to better determine directionality of the measured associations. Research should also examine the associations of mental health with HIV acquisition and disease progression with a wider range of conflict-affected populations, as the relationship may vary depending on the population and the possibilities for risk exposure.

## Conclusions

Existing literature demonstrates that depression, anxiety, and PTSD have been quantifiably associated with four factors related to HIV acquisition and disease progression in conflict-affected populations: markers of HIV risk (i.e. STIs), HIV-related health status (e.g. CD4 count), sexual risk behaviors, and HIV risk exposures (i.e. sexual violence). Specifically, poor mental health has been associated with two outcomes, HIV markers and sexual risk behaviors, while HIV risk exposures and health status have been associated with the outcome of poor mental health. Additional research utilizing random selection and longitudinal design can further establish the strength of these associations and determine if HIV and mental health services need to be integrated for conflict-affected populations.

## Additional file

Additional file 1: Scoping review search terms for PubMed. (DOCX 14 kb)

## Abbreviations

AOR: Adjusted Odds Ratio
ART: Antiretroviral Therapy
CI: Confidence Interval
HIV: Human Immunodeficiency Virus
HTQ: Harvard Trauma Questionnaire
IDP: Internally Displaced Person
MDD: Major Depressive Disorder
OR: Odds Ratio
PTSD: Posttraumatic Stress Disorder
SD: Standard Deviation
SE: Standard Error
STI: Sexually Transmitted Infection
UNESCO: United Nations Educational, Scientific and Cultural Organization
USA: United States of America
