# Supplementary material for: A scoping review of the associations between mental health and factors related to HIV acquisition and disease progression in conflict-affected populations
Source: Confl Health. 2018 Jun 1;12:20. doi: 10.1186/s13031-018-0156-y (PMC5984364; doi:10.1186/s13031-018-0156-y)
Supplement: Supplementary file 1 — Scoping review search terms for PubMed. (DOCX 14 kb) [file 13031_2018_156_MOESM1_ESM.docx]

Additional file 1: Scoping review search terms for PubMed

**Concept 1**

‘Sexual risk behavior’[TIAB] OR (‘Risk Reduction Behavior’[Mesh] OR ‘Risk Reduction Behavior’[TIAB] OR ‘Risk Reduction Behaviors’[TIAB] OR ‘Risk’[TIAB])

AND (HIV[TIAB] OR AIDS[TIAB] OR ‘Human Immunodeficiency Virus’[TIAB] OR ‘Acquired Immune Deficiency’[TIAB])

OR

‘sexually transmitted infection’[Mesh] OR ‘sexually transmitted infection’[TIAB] OR ‘sexually transmitted infections’[TIAB] OR ‘sexually transmitted infections’[Mesh] OR ‘STI’[TIAB] OR ‘STI’[Mesh] OR ‘STIs’ [TIAB] OR ‘STIs’ [Mesh] OR ‘sexually transmitted disease’[Mesh] OR ‘ sexually transmitted disease’[TIAB] OR ‘sexually transmitted diseases’[TIAB] OR ‘sexually transmitted diseases’[Mesh] OR ‘STD’[TIAB] OR ‘STD’[Mesh] OR ‘STDs’[TIAB] OR ‘STDs’[Mesh] OR ‘sexual infection’[TIAB] OR ‘sexual infection’[Mesh] OR ‘sexual infections’[TIAB] OR ‘sexual infections’[Mesh] OR ‘sexual disease’[TIAB] OR ‘sexual disease’[Mesh] OR ‘sexual diseases’[TIAB] OR ‘sexual diseases’[Mesh]

**Concept 2**

AND

‘Mental health’[TIAB] OR ‘Mental Health’[Mesh] OR ‘Stress Disorders, Post-Traumatic’[Mesh] OR ‘Post-Traumatic Stress Disorders’[TIAB] OR ‘Post-Traumatic Stress Disorder’[TIAB] OR ‘Anxiety’[Mesh] OR hypervigilance[TIAB] OR nervousness[TIAB] OR ‘Depression’[Mesh] OR depressive[TIAB] OR depressed[TIAB] OR PTSD[TIAB] OR psychological[TIAB] OR psychosocial[TIAB] OR psychiatric[TIAB]

*Concept 3 (NOT internal conflict)*

AND

War[TIAB] OR ‘conflict setting’[TIAB] OR ‘post-conflict’[TIAB] OR ‘post conflict’[TIAB] OR ‘armed conflict’[TIAB] OR ‘internal conflict’[TIAB] OR ‘conflict affected’[TIAB] OR ‘non-state conflict’ [TIAB] OR genocide[TIAB]
